# TRAINING AT WORK: THE IMPACT OF INTERNALIZED AGEISM

**DOI:** 10.1093/geroni/igz038.010

**Published:** 2019-11-08

**Authors:** Sarah A Vickerstaff, Mariska F van der Horst

**Affiliations:** 1 University of Kent, Canterbury, Kent, United Kingdom; 2 Vrije Universiteit Amsterdam, Amsterdam, Noord-Holland, Netherlands

## Abstract

It is well documented that seniors receive less work based training than younger counterparts. Studies have assessed the extent to which managers afford access to training because of stereotypes about older workers being less trainable or interested in training. Less is known about the degree to which individuals have internalised age stereotypes and self-stereotype which impacts their attitudes about training. In this work place based study, through analysis of interviews with older workers we examine the extent to which internalised ageism is an inhibitor for seeking further training. We examine the role of gender and work setting. The data includes 185 participants in five different UK organisations in blue collar, white collar, managerial, manufacturing and services sectors. There is some evidence that older workers may feel that they are less deserving and less capable of training and development with implications for policy in this field.

